# Expression of nuclear retinoid receptors in normal, premalignant and malignant gastric tissues determined by in situ hybridization

**DOI:** 10.1038/sj.bjc.6690340

**Published:** 1999-04

**Authors:** S-Y Jiang, S-R Shen, R-Y Shyu, J-C Yu, H-J Harn, M-Y Yeh, M M-S Lee, Y-C Chang

**Affiliations:** Graduate Institute of Medical Sciences, Department of Microbiology and Immunology, School of Public Health, National Defense Medical Center, Taipei, Taiwan; Departments of Medical Research, Internal Medicine, Surgery, Pathology, Tri-Service General Hospital, Taipei, Taiwan; Department of Mathematics, Tamkang University, Taipei, Taiwan, Republic of China

**Keywords:** gastric cancer, retinoic acid receptor, retinoid x receptor, in situ hybridization, mRNA

## Abstract

Retinoids exhibit multiple functions through interaction with nuclear retinoid receptors and have growth-suppressive activity on gastric cancer cells. To better understand the roles of nuclear retinoid receptors during gastric carcinogenesis, we have used in situ hybridization to investigate expression of retinoic acid receptors (RARs) and retinoid x receptors (RXRs) in premalignant and malignant formalin-fixed paraffin-embedded gastric tissues. Histological sections of eight normal, 17 distal normal and nine gastric cancer tissues were hybridized with non-radioactive RNA probes for subtypes of RAR and RXR. Expression of RARα, RARβ, RARγ, RXRα and RXRβ was found in most cell types in gastric mucosa tissues from normal individuals as well as in distal normal tissues from cancer patients. Expression of RARα and RARβ were found in three and seven cancer tissues, respectively, and levels of RXRα mRNA were significantly decreased in poorly differentiated cancer tissues. Among the five investigated nuclear retinoid receptors, only expression of RARα mRNA was significantly decreased in intestinal metaplasia, dysplasia and cancer tissues when compared to adjacent normal tissues. In conclusion, normal gastric mucosa expressed both RARs and RXRs, which supports the physiological role of retinoic acid on normal gastric mucosa. The decrease in RARα expression in premalignant and malignant gastric tissues suggests a significant role of RARα during gastric carcinogenesis. © 1999 Cancer Research Campaign


*British Journal of Cancer* (1999) **80**(1/2), 206–214
© 1999 Cancer Research Campaign
Article no. bjoc.1998.0340

### Expression of nuclear retinoid receptors in normal, premalignant and malignant gastric tissues determined by in situ hybridization

**S-Y Jiang[1,4], S-R Shen[2,4], R-Y Shyu[5], J-C Yu[6], H-J Harn[7], M-Y Yeh[2,4], MM-S Lee[3] and Y-C Chang[8]**

[1]Graduate Institute of Medical Sciences, [2]Department of Microbiology and Immunology, [3]School of Public Health, National Defense Medical Center, Taipei, Taiwan, Departments of [4]Medical Research, [5]Internal Medicine, [6]Surgery, [7]Pathology, Tri-Service General Hospital, Taipei, Taiwan; [8]Department of Mathematics, Tamkang University, Taipei, Taiwan, Republic of China

**Summary** Retinoids exhibit multiple functions through interaction with nuclear retinoid receptors and have growth-suppressive activity on gastric cancer cells. To better understand the roles of nuclear retinoid receptors during gastric carcinogenesis, we have used in situ hybridization to investigate expression of retinoic acid receptors (RARs) and retinoid x receptors (RXRs) in premalignant and malignant formalin-fixed paraffin-embedded gastric tissues. Histological sections of eight normal, 17 distal normal and nine gastric cancer tissues were hybridized with non-radioactive RNA probes for subtypes of RAR and RXR. Expression of RARα, RARβ, RARγ, RXRα and RXRβ was found in most cell types in gastric mucosa tissues from normal individuals as well as in distal normal tissues from cancer patients. Expression of RARα and RARβ were found in three and seven cancer tissues, respectively, and levels of RXRα mRNA were significantly decreased in poorly differentiated cancer tissues. Among the five investigated nuclear retinoid receptors, only expression of RARα mRNA was significantly decreased in intestinal metaplasia, dysplasia and cancer tissues when compared to adjacent normal tissues. In conclusion, normal gastric mucosa expressed both RARs and RXRs, which supports the physiological role of retinoic acid on normal gastric mucosa. The decrease in RARα expression in premalignant and malignant gastric tissues suggests a significant role of RARα during gastric carcinogenesis.

**Keywords:** gastric cancer; retinoic acid receptor; retinoid x receptor; in situ hybridization; mRNA

Retinoids, compounds exhibiting diverse biological activities, are associated with immunological activation, vision, differentiation and cancer growth control. Their effects on gene expression are mediated through binding with nuclear retinoic acid receptor (RAR) and retinoid x receptor (RXR) (Mangelsdorf et al, 1994; Leblanc and Strunnenberg, 1995). Three subtypes of RAR and RXR (α, β and γ) and multiple isoforms within each subtype of receptor have been identified. Each receptor subtype exhibits distinct functions, which is supported by their specific patterns of expression in the developing mouse embryo as well as unique distributions in adult tissues (Mangelsdorf et al, 1994). Both RAR and RXR share high sequence homology in the DNA and ligand-binding domains. RARs bind all-*trans* retinoic acid (RA) and 9-*cis* RA with high affinity; whereas RXRs bind with high affinity only with the 9-*cis* RA (Heyman et al, 1992). RA-mediated regulation of gene expression is primarily acted through the formation of RAR/RXR heterodimers that bind with high affinity to the DNA sequence known as the retinoic acid response element. In addition to forming heterodimers with RAR, RXR can also form homodimers, in the presence of 9-*cis* RA, or heterodimers with thyroid hormone receptor, vitamin D receptor or orphan receptors (Mangelsdorf et al, 1994; Leblanc and Strunnenberg, 1995).

The nuclear receptors appear to be the prominent mediators of RA action on gene expression. Therefore, it is of interest to determine whether their levels are altered during cancer development and whether their expression patterns in premalignant and malignant tissues can provide prognostic information. Cancer cell lines are found to have lost or have alterations in RARβ expression (Hu et al, 1991; Shyu et al, 1995). In addition, the introduction of RARβ into cells which do not express RARβ leads to suppression of tumorigenesis in vivo (Houle et al, 1993; Kaiser et al, 1997). These results suggest that RARβ may function as a tumour suppressor. The frequent loss of RARβ expression in cancer tissues from the head and neck, lung and breast supports this conclusion (Xu et al, 1994*a*, 1997*a*, 1997*b*; Widschwendter et al, 1997). Down-regulation of RARα was observed in chemical-induced as well as H-*ras*-transformed mouse skin tumours (Darwiche et al, 1995, 1996) and squamous head and neck tumours (Issing and Wustrew, 1996). Decreased RARγ expression was observed in mouse skin tumours (Darwiche et al, 1995, 1996) and non-small-cell lung tumours (Xu et al, 1997*a*). In addition, RXRα was found to decrease in mouse skin tumours (Darwiche et al, 1995) and RXRβ was found to increase in non-small-cell lung tumours (Xu et al, 1997*a*). These results suggest each subtype of

*Received 27 April 1998*
*Revised 27 July 1998*
*Accepted 10 August 1998*

*Correspondence to:* S-Y Jiang, PO Box 90048-518, Graduate Institute of Medical Sciences, National Defense Medical Center, Taipei, Taiwan 100, Republic of China nuclear retinoid receptor may be altered distinctly in different tissues during carcinogenesis. This is further evidence that each receptor subtype has a unique function in different tissues.

The role of retinoids in gastric cancer treatment and prevention has been studied previously. Epidemiological and animal studies have demonstrated the activities of retinoids in prevention (Haenszel et al, 1985; Miasoedov et al, 1989) and treatment (Fujii et al, 1991) of gastric cancer. These studies support our recent observation of the growth suppressive activity of all *trans* RA and 13-*cis* RA on gastric cancer cells in vitro and in vivo (Shyu et al, 1995; Jiang et al, 1996). To further understand the roles of nuclear retinoid receptors in gastric mucosa, we have used an in situ hybridization method to detect expression of subtypes of RAR and RXR in histological sections of formalin-fixed, paraffin-embedded gastric tissues from normal individuals and gastric cancer patients.

### MATERIALS AND METHODS

### Specimen collection and preparation

Four pairs of biopsy specimens from the gastric body and antrum regions of normal individuals undergoing health check-ups were obtained using a pandoscope. In addition, nine gastric cancer tissues and 17 distal normal tissues (eight from the body and nine from the antrum regions) from nine patients were obtained by pandoscope. Tissues were fixed in 10% neutral formalin and embedded in paraffin. The specimens were sliced into 4-μm-thick sections. Haematoxylin and eosin (H&E)-stained tissue sections were screened by the same pathologist to identify adjacent normal tissues, intestinal metaplasia, dysplasia and carcinoma. Gastric cancer tissues were classified based on the criteria of World Health Organization (Watanabe et al, 1989).

### Subcloning of RAR and RXR cDNA

The 1.9 kb of human RARα (Petkovich et al, 1987), 1.9 kb of human RXRα (Mangelsdorf et al, 1990) and 2.2 kb of human RXRβ (Hamada et al, 1989) cDNA fragments were cloned into the Eco RI site of the plasmid pBSK$^{+/-}$ (Stratagene, La Jolla, CA, USA) through blunt end ligation. The 1.6 kb of human RARβ (Brand et al, 1988) and 1.7 kb of human RARγ (Zelent et al, 1989) cDNA fragments were cloned into the Not I site of the plasmid pRC/CMV (Invitrogene Co., San Diego, CA, USA) through blunt end ligation.

### Preparation of digoxigenin-labelled RNA probes

The digoxigenin-labelled RNA probes spanning the entire open-reading frames of cDNAs of RARα, RARβ, RARγ, RXRα or RXRβ cDNA were synthesized using an in vitro transcription kit obtained from Boehringer Mannheim (Germany) (Xu et al, 1994*b*). The RNA probes were precipitated, washed and dissolved in diethylpyrocarbonate-treated water containing RNAsin. The concentration was adjusted to 100 ng ml$^{-1}$ using a DNA Dipstick Kit (Invitrogene) and stored at –70°C. The length of the RNA probes was confirmed by RNA gel electrophoresis followed by visualization using the fluorescence substrate CSPD as described below. The specificity of the probes was determined by Northern blotting.

### RNA isolation and Northern blotting

SC-M1 and TSGH9201 human gastric cancer cell lines cells were grown in T75 cm$^2$ flasks in RPMI-1640 medium supplemented with 5% fetal bovine serum and antibiotics in a humidified atmosphere of 5% carbon dioxide and 95% air at 37°C. Polyadenosine (poly-A)$^+$ RNA was purified from cellular lysates using oligo dT cellulose as described by Badley and co-workers (Badley et al, 1987). RNA was then fractionated on a 1.1% agarose, 1.1% formaldehyde gel in 5 mM NaOAc, 1 mM EDTA, 20 mM 3-[*N*-morpholino]propanesulphonic acid, pH 7.0 and transferred to a nylon membrane (Boehringer Mannheim) by capillary blotting in 20 × saline–sodium citrate (SSC; 3 M NaCl, 0.3 M $Na_3$ citrate, pH 7.0). Blots were UV-fixed, prehybridized and hybridized at 68°C in buffer containing 50% (v/v) formamide, 5 × SSC, 2% (w/v) blocking reagent (Boehringer Mannheim), 0.1% *N*-lauroyl-sarcosine and 0.2% (w/v) sodium dodecyl sulphate (SDS). The membranes were washed first with 2 × SSC containing 0.1% SDS and then washed with 0.1 × SSC containing 0.1% SDS at 68°C for 30 min. Specific hybridization was detected by a DIG luminescence detection kit using CSPD as the substrate and was recorded using Kodak XAR-5 film at room temperature. Probes were removed and membranes were then hybridized with the digoxigenin-labelled probe for glyceraldehyde-3-phosphate dehydrogenase (GAPDH).

### In situ hybridization

A nonradioactive in situ hybridization using digoxigenin-labelled riboprobes was used as described by Xu and co-workers (Xu et al, 1994*b*). Briefly, sections were deparaffinized, rehydrated and deproteinized by digestion with protease K. The slides were fixed with 4% paraformaldehyde and then acetylated in 0.25% acetic anhydride in 0.1 M triethanolamine-HCl buffer. After washing and dehydration, the slides were prehybridized for more than 1 h at 42°C with a hybridization solution containing 50% deionized formamide, 2 × SSC, 2 × Denhardt's solution (0.02% Ficoll 400, 0.02% polyvinylpyrrolidone, 0.02% bovine serum albumin), 10% dextran sulphate, 400 μg ml$^{-1}$ yeast tRNA, 250 μg ml$^{-1}$ salmon sperm DNA and 20 mM dithiothreitol in diethylpyrocarbonate-treated water. Then, the slides were incubated with a 50 μl per slide hybridization solution containing 100 ng freshly denatured digoxigenin-labelled RNA probes at 42°C for 16 h in closed humid containers. After incubating with RNase A, the slides were washed and then blocked for non-specific binding using 2% normal sheep serum. The slides were incubated overnight at 4°C with sheep anti-digoxigenin antibody, and the in situ hybridization signal was visualized by incubating the slides in a chromogen solution containing nitroblue tetrazolium and X-phosphate (Boehringer Mannheim). Slides were observed under an AH-1 light microscope (Olympus, Japan) and then stored dark at 4°C.

### Reviewing and scoring the sections

All sections from the same patient were stained on the same day with the same reagents to ensure a proper comparison of the different sections. The sections were reviewed by three independent researchers, including a pathologist. The staining of the sections was assigned scores ranging from 0 to 4, representing no staining (0), weak (1 +), positive (2 +), strongly positive (3 +), or very strongly positive (4 +). The intensity of staining in the surface

© Cancer Research Campaign 1999

mucus cells, mucus neck cells, parietal cells and chief cells was analysed in the body tissues. The intensity of staining in the surface mucus cells and regenerative and mature gland cells in the antrum tissues was analysed. Correlation of RXRα and RXRβ expression between cancer tissues with poor vs non-poor differentiation was analysed by Fisher's Exact Test. Differential expression of RARα mRNA in adjacent normal, intestinal metaplasia adjacent to gastric cancer, dysplasia adjacent to gastric cancer and gastric cancer tissues from different or the same gastric cancer patients was analysed by logistic regression. However, the estimated coefficients and their correspondent variances were obtained through the generalized estimating equations method (Liang and Zeger, 1986) and the SAS/IML macro program, GEE1.SAS, to take into account the within subject correlation.

### RESULTS

### Activity and specificity of digoxigenin-labelled RNA probes

The activity of digoxigenin-labelled probes was first tested by Northern hybridization on gastric cancer cells. Antisense RNA probes of each RAR or RXR subtype could detect specific mRNA subtypes of RAR and RXR (Figure 1). The molecular weights of each subtype of RAR or RXR mRNA transcript in SC-M1 or TSGH9201 gastric cancer cells detected were similar to results described previously (Shyu et al, 1995). Therefore, the binding of antisense RNA probes was specific. No band was shown in the mRNA samples hybridized with five control sense RNA probes.

### Staining of RAR and RXR mRNA in tissues

Expression of the mRNAs for RAR and RXRs in gastric cancer tissues was analysed by non-radioactive in situ hybridization. Figure 2 shows the mRNA localization of RARα, RARβ, RARγ, RXRα and RXRβ in consecutive sections of a specimen from the body region with moderately differentiated gastric cancer by digoxigenin-labelled RNA probes. Positive results appeared as a dark purple colour in the cytoplasm, where mRNA is expected to be localized. The adjacent normal gland had the highest expression of RXRα mRNA and the lowest expression of RARβ mRNA (see below). The sense probes did not bind to the adjacent sections, indicating that hybridization with the antisense probes was specific.

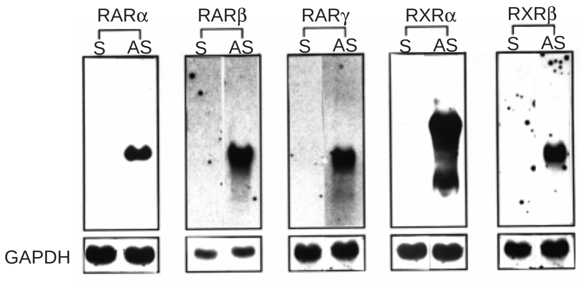

**Figure 1** Northern blot analysis of RAR and RXR in gastric cancer cells. mRNA from SC-M1 cells was hybridized with sense (S) or antisense (AS) RNA probes for RARα, RARγ, RXRα or RXRβ. mRNA from TSGH9201 cells was hybridized with RARβ sense and antisense RNA probes. Probes were removed and the same membranes were then hybridized with a GAPDH probe

### Expression of RARs and RXRs in non-malignant gastric body and antrum tissues

Four pairs of body and antrum tissues from individuals without cancer were analysed for in situ expression of nuclear retinoid receptor mRNAs. H&E staining showed that three specimens exhibited features of gastritis and one exhibited atrophic gastritis in both antrum and body tissues. In addition to gastritis, intestinal metaplasia and dysplasia were also found in two antrum tissues. Expression of RAR and RXR mRNAs in areas without intestinal metaplasia and dysplasia was analysed.

Expression of mRNAs of nuclear retinoid receptors in surface mucus, mucus neck, parietal and chief cells was analysed in specimens derived from the body tissues. In the antrum tissues, expression of nuclear retinoid receptors in surface mucus, regenerative gland and mature gland cells was analysed. Most cell types analysed from both body and antrum tissues expressed five subtypes of nuclear retinoid receptors (data not shown). No cell type-specific expression of a unique subtype of RAR or RXR was observed. However, mRNA of RXRα was found to express at levels equivalent to or higher than that of the other four tested nuclear retinoid receptors in all analysed cell types. Also, RARα mRNA appeared to be more abundant than RARβ or RARγ in some specimens. Similar results were obtained in eight distal normal body and nine distal normal antrum tissues from patients with gastric cancer.

### Expression of RARs and RXRs in gastric cancer tissues

Nine gastric cancer specimens, four derived from the body region and five derived from the antrum region, were analysed. All tissues exhibited the morphology of adenocarcinoma. Two specimens exhibited the morphology of both tubular and papillary tumours. Four specimens were the tubular type and three were the signet-ring cell type (Table 1). Among nine investigated cancer tissues, RARγ, RXRα and RXRβ were expressed in all nine, while RARα and RARβ were expressed in three and seven cancer tissues respectively. Among tissues found to express RARα, two were well-differentiated and one was moderately differentiated. Both papillary and tubular types of adenocarcinoma cells were found to stain positively for RARα mRNA in the two cancer tissues that exhibited the morphology of both. None of two cancer tissues with poor differentiation expressed RARα. Among six tissues without RARα expression, three exhibited the morphology of tubular type and the other three were the signet-ring cell type tumours. Well and moderately differentiated gastric adenocarcinoma expressed higher levels of RXRα mRNA than the poorly differentiated adenocarcinoma, and the difference was significant ($P = 0.03$). A similar trend, although not a significant difference ($P = 0.083$), was observed for RXRβ.

### Differential expression of RARs and RXRs in distal normal tissues, intestinal metaplasia adjacent to gastric cancer, and dysplasia adjacent to gastric cancer tissues from different gastric cancer patients

Adjacent normal tissues, intestinal metaplasia, dysplasia and cancer tissues from the same tissue section of different gastric cancer patients were compared. Most of the gastric cancer cells were believed to be derived from neck cells which exhibited

© Cancer Research Campaign 1999

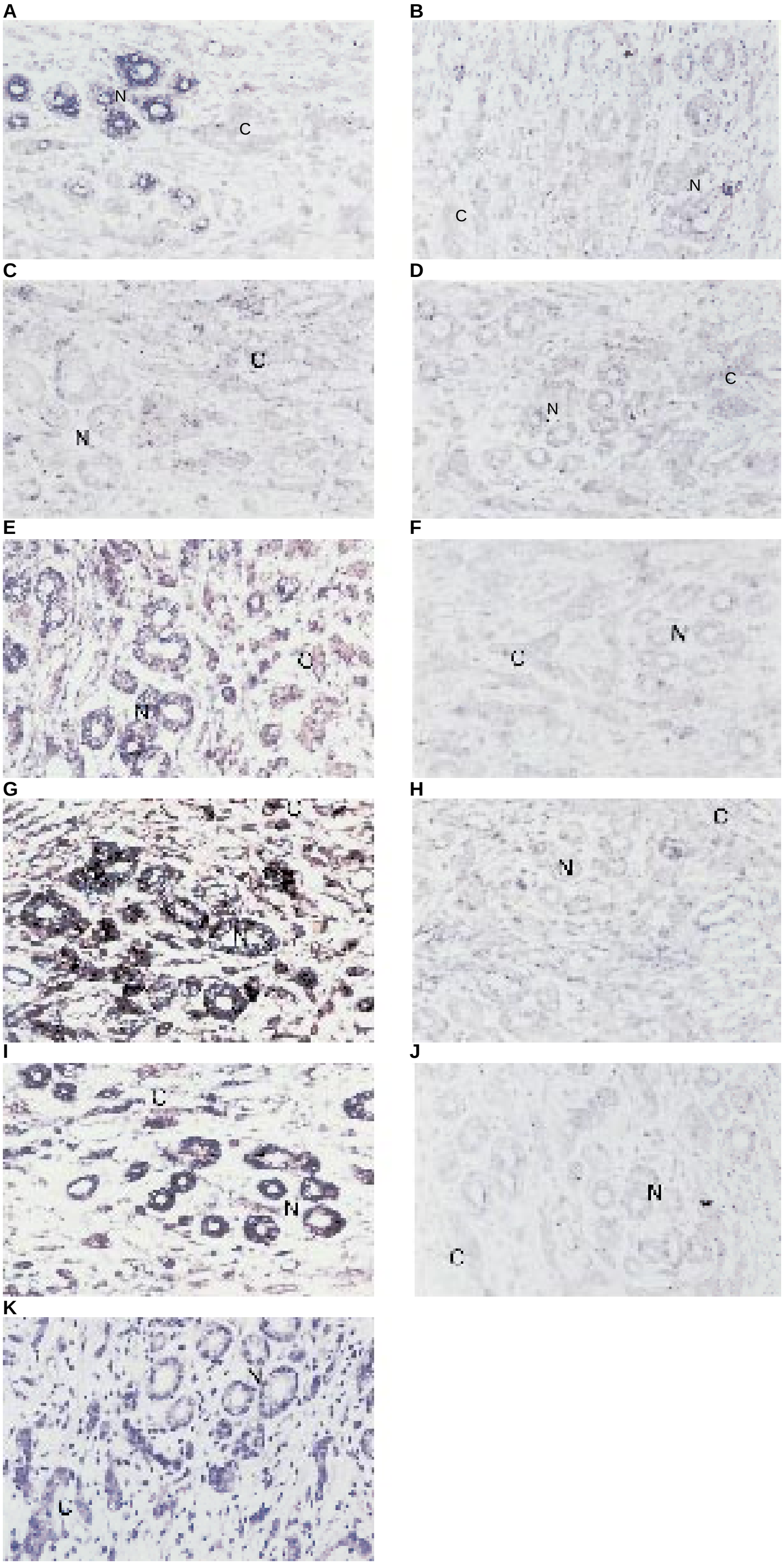

**Figure 2** Detection of subtypes of RAR and RXR mRNA expression in a gastric cancer tissue with moderately differentiated adenocarcinoma from the body region using RNA probes. Tissues were hybridized with antisense (**A, C, E, G** and **I**) or sense (**B, D, F, H** and **J**) probes for RARα (**A** and **B**), RARβ (**C** and **D**), RARγ (**E** and **F**), RXRα (**G** and **H**) and RXRβ (**I** and **J**) respectively. (**K**) H&E staining. Magnification 100 ×; N, adjacent normal tissues; C, cancer tissues

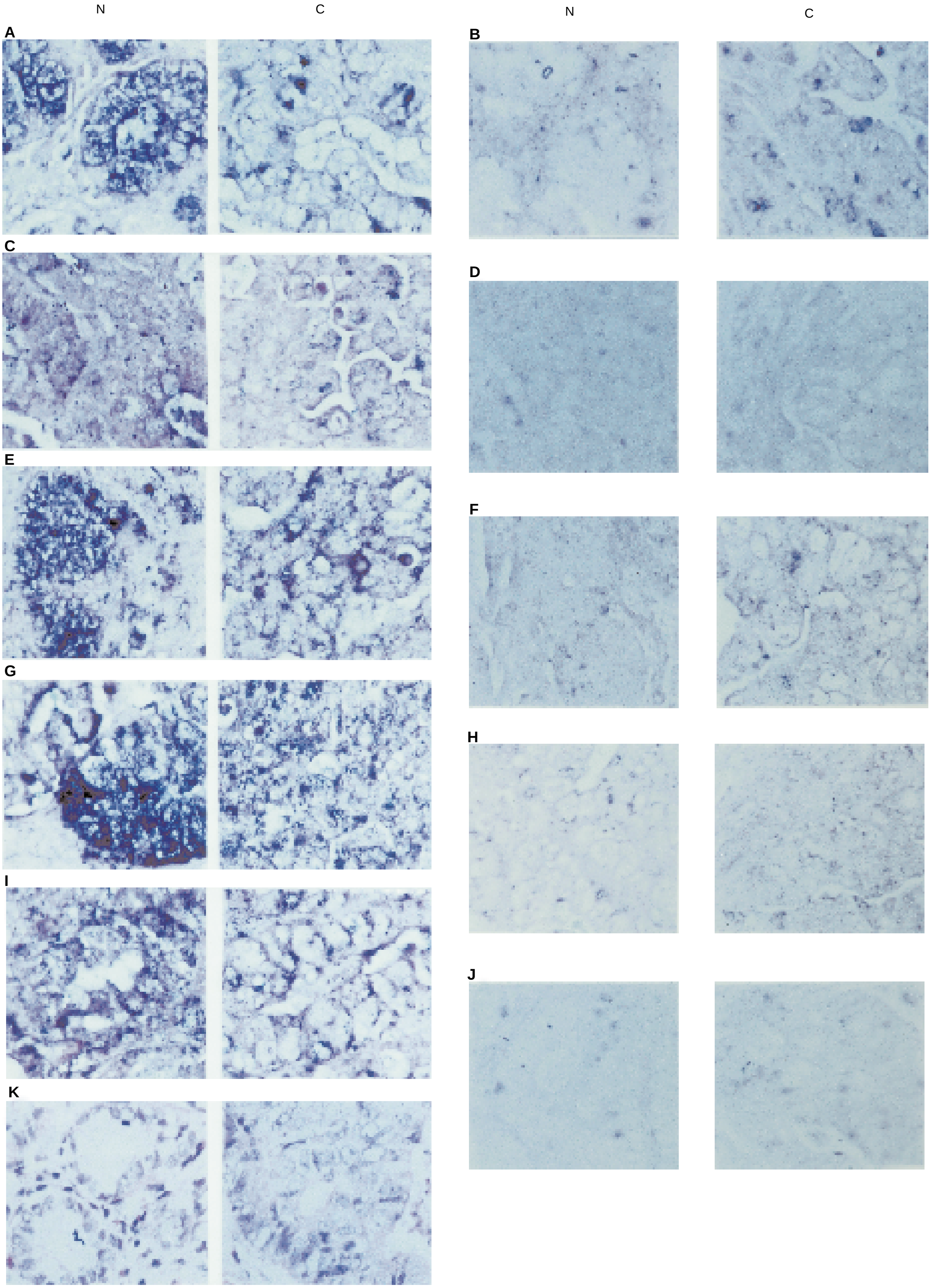

**Figure 3** Detection of subtypes of RAR and RXR mRNA expression in a gastric cancer tissue with well-differentiated adenocarcinoma from the antrum region using RNA probes. Tissues were hybridized with antisense (**A, C, E, G** and **I**) or sense (**B, D, F, H** and **J** probes for RARα (**A** and **B**), RARβ (**C** and **D**), RARγ (**E** and **F**), RXRα (**G** and **H**) and RxRβ (**I** and **J**) respectively. (**K**) H&E staining. The adjacent normal tissues (N) are shown on the left of each panel and cancer lesions (C) are shown on the right. Magnification × 200

 *© Cancer Research Campaign 1999*

**Table 1** Expression of mRNA of nuclear retinoid receptors in gastric cancer tissues

| Case no. | Differentiation | Histological types | RARα | RARβ | RARγ | RXRα | RXRβ |
|---|---|---|---|---|---|---|---|
| 1 | Well | Tubular + papillary | +* | ++ | +++ | ++++ | ++ |
| 2 | Well | Tubular + papillary | + | ++ | ++ | ++++ | ++ |
| 3 | Well | Tubular | – | + | ++ | +++ | ++ |
| 4 | Moderate | Tubular | ++ | ++ | +++ | +++ | +++ |
| 5 | Moderate | Signet-ring cell | – | – | ++ | +++ | +++ |
| 6 | Moderate | Tubular | – | – | ++ | +++ | + |
| 7 | Moderate | Tubular | – | ++ | + | ++++ | ++ |
| 8 | Poor | Signet-ring cell | – | + | + | ++ | + |
| 9 | Poor | Signet-ring cell | – | + | ++ | ++ | + |

[a]Intensity of staining. –, no staining; +, weak staining; ++, positive staining; +++, strongly positive staining, ++++, very strongly positive staining.

**Table 2** Differential expression of RARs and RXRs in distal normal, adjacent normal, intestinal metaplasia adjacent to gastric cancer and dysplasia adjacent to gastric cancer from different gastric cancer patients

| Receptors | % Positive[a] (No. positive/total) | | | | |
|---|---|---|---|---|---|
| | Distal normal[b] | Adjacent normal[b] | IM[c] | Dysplasia | Cancer |
| RARα | 100 (17/17) | 88 (7/8)[d,e,f] | 43 (3/7)[d] | 38 (3/8)[e] | 25 (2/8)[f] |
| RARβ | 100 (17/17) | 88 (7/8) | 86 (6/7) | 88 (7/8) | 88 (7/8) |
| RARγ | 100 (17/17) | 100 (8/8) | 100 (7/7) | 100 (8/8) | 100 (8/8) |
| RXRα | 100 (17/17) | 100 (8/8) | 100 (7/7) | 100 (8/8) | 100 (8/8) |
| RXRβ | 100 (17/17) | 100 (8/8) | 100 (7/7) | 100 (8/8) | 100 (8/8) |

[a]Positive cases were those stained with an intensity score of > 1. [b]Intensity of staining in mucous neck cells or regenerative antrum gland cells. [c]Intestinal metaplasia. [d]$P < 0.05$; odds ratio (OR) = 0.11; 95% confidence interval (CI), (0.01, 0.88). [e]$P < 0.05$; OR = 0.09; 95% CI, (0.01, 0.75). [f]$P < 0.05$; OR = 0.05; 95% CI, (0.00, 0.50).

**Table 3** Expression of RARs and RXRs in adjacent normal tissues, intestinal metaplasia, dysplasia and cancer lesions from the same gastric cancer patient

| Receptors | No. of cases | No. of cases (% of total) | | |
|---|---|---|---|---|
| | | AT > C[b] | AT = C[c] | AT < C[d] |
| Comparison of cancer and adjacent normal tissues[a] | | | | |
| RARα | 8 | 7 (88)[e,f] | 1 (12) | 0 (0) |
| RARβ | 8 | 0 (0) | 8 (100) | 0 (0) |
| RARγ | 8 | 2 (15) | 6 (75) | 0 (0) |
| RxRα | 8 | 0 (0) | 8 (100) | 0 (0) |
| RxRβ | 8 | 3 (37) | 5 (63) | 0 (0) |
| Comparison of cancer and intestinal metaplasia tissues | | | | |
| RARα | 7 | 2 (29)[e] | 4 (57) | 1 (14) |
| RARβ | 7 | 0 (0) | 7 (100) | 0 (0) |
| RARγ | 7 | 0 (0) | 7 (100) | 0 (0) |
| RxRα | 7 | 0 (0) | 7 (100) | 0 (0) |
| RxRβ | 7 | 1 (14) | 5 (72) | 1 (14) |
| Comparison of cancer and adjacent dysplasia tissues | | | | |
| RARα | 8 | 2 (25)[f] | 5 (63) | 1 (12) |
| RARβ | 8 | 0 (0) | 8 (100) | 0 (0) |
| RARγ | 8 | 0 (0) | 8 (100) | 0 (0) |
| RxRα | 8 | 0 (0) | 8 (100) | 0 (0) |
| RxRβ | 8 | 1 (13) | 7 (88) | 0 (0) |

[a]Compared with adjacent mucus neck cells in body tissues or adjacent regenerative gland in antrum tissues. [b]A > C: expression of mRNA was greater in adjacent tissues (AT) than in cancer (C). [c]A = C: expression of mRNA was similar in adjacent tissues (AT) than in cancer (C). [d]A < C: expression of mRNA was lower in adjacent tissues (AT) than in cancer (C). [e]$P < 0.05$; odds ratio (OR) = 0.06; 95% confidence Interval (CI), (0.01, 0.57). [f]$P < 0.05$; OR = 0.02, 95% CI, (0.00, 0.19).

© Cancer Research Campaign 1999

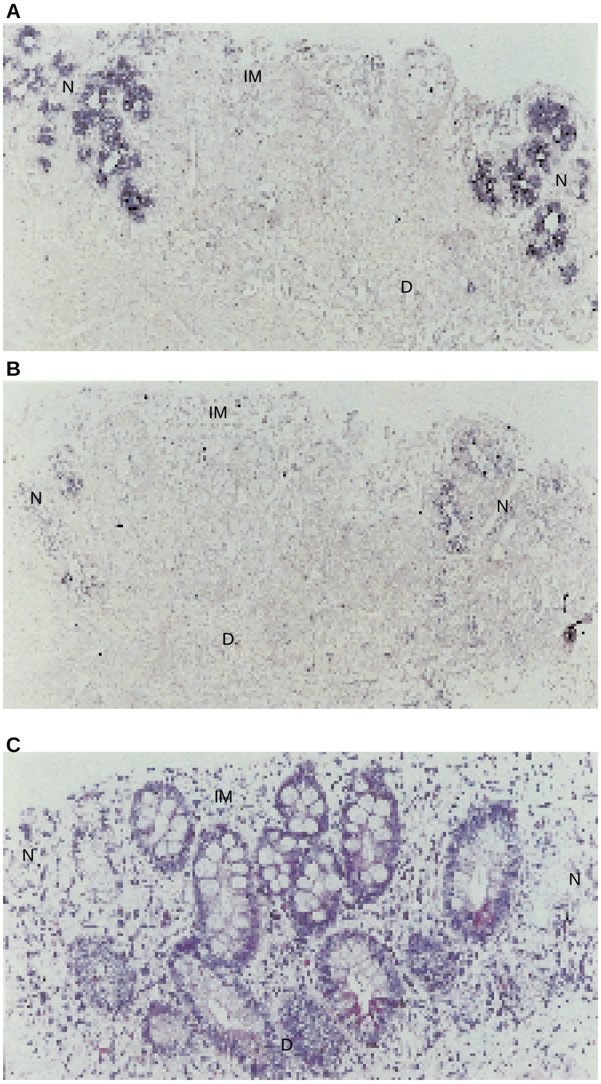

**Figure 4** Detection of RARα mRNA in a non-malignant mucosa tissue from the body region of a patient with well-differentiated gastric adenocarcinoma in the antrum. The morphology of atrophic gastritis is shown by H&E staining (bottom). Tissue sections were hybridized with antisense (**A**) or sense (**B**) RARα probes. Normal glands (N), intestinal metaplasia (IM) or dysplasia (D) tissues are indicated. Magnification × 200

highly proliferative potential. Therefore, mucus neck cells of the body tissues or the regenerative gland cells of the antrum tissues were chosen to compare to pre-neoplastic and cancer cells in levels of RAR and RXR expression. Representative staining of the tissues containing adjacent normal tissues as well as moderately differentiated or well-differentiated adenocarcinoma is shown in Figure 2 and Figure 3 respectively. In Figure 2, mucus neck cells in the tissue expressed RARα with an intensity of '++' (Figure 2A). However, the adjacent cancer cells did not express RARα. RARβ was not expressed in either normal or cancer cells (Figure 2C). RARγ was expressed in both normal and cancer cells with an intensity of '++' (Figure 2E). RXRα and RXRβ were expressed in both normal and cancer cells with an intensity of '++++' in the adjacent tissues and '+++' in the cancer tissues (Figure 2G, I). Similarly, adjacent normal and cancer tissues from a specimen with well-differentiated gastric adenocarcinoma were stained positive for RARβ, RARγ, RXRα and RXRβ mRNAs (Figure 3). RARα mRNA was expressed in the adjacent normal tissue. However, it was not expressed in the cancer tissues. Figure 4 shows the representative RARα mRNA staining of the atrophic gastritis tissue with the morphology of normal glands, intestinal metaplasia and dysplasia. Expression of RARα was found only in adjacent normal glands but not in intestinal metaplasia and dysplasia.

Table 2 summarizes the frequency of RAR and RXR mRNA expression from eight cancer tissues. One cancer tissue was not analysed due to a lack of adjacent non-malignant tissue. Mucus neck cells from body tissues and regenerative gland cells from the antrum tissues of normal individuals described above, and distal normal tissues obtained from cancer patients, expressed all five nuclear retinoid receptors (Table 2). In the cancer tissues, only two among eight specimens stained positive for RARα mRNA. Frequency of RARα expression in tissues with intestinal metaplasia, dysplasia or cancer was significantly lower than in the adjacent normal tissues ($P < 0.05$; Table 2). Loss of RARα expression was correlated with an increase in the morphological features from normal to cancer. Only one specimen did not have RARβ expression in the adjacent normal tissues, intestinal metaplasia, dysplasia and cancerous tissues. All eight cancer specimens with all histological features expressed RARγ, RXRα and RXRβ.

### Expression of RARs and RXRs in adjacent normal tissue, intestinal dysplasia and cancer lesions from the same gastric cancer patient

The expression levels of RARs and RXRs in normal, premalignant and malignant tissues within the same slide section are compared (Table 3). Levels of RARα mRNA in adjacent normal, intestinal metaplasia and dysplasia tissues were found to be higher than those in cancer tissues in seven, two and two cases respectively. Significantly more cases of adjacent normal tissues than intestinal metaplasia and dysplasia had levels of RARα mRNA expression higher than that of cancer cells ($P < 0.05$). No similar result was found for the other four nuclear retinoid receptors. mRNA levels of RARβ and RXRα in adjacent normal, intestinal metaplasia and dysplasia tissues were similar to cancer lesions in all cases. RARγ and RXRβ mRNA expression in adjacent normal tissues in two and three cases, respectively, was found to be greater than that in cancer lesions. However, most intestinal metaplasia, dysplasia and cancer lesions had similar levels of RaRγ mRNA.

### DISCUSSION

This study analysed in situ expression of nuclear retinoid receptors in premalignant and malignant gastric tissues. Normal gastric mucosa tissues expressed all five nuclear retinoid receptors, and levels of RXRα mRNA were the highest among the five investigated nuclear retinoid receptors in both normal gastric mucosa and cancer tissues. No differences in expression of RARβ, RARγ, RXRα and RXRβ between normal tissues and cancer lesions were observed. However, expression of RARα was significantly decreased in intestinal metaplasia, dysplasia and cancer tissues.

Our data showed that tissues of normal gastric mucosa from the body and antrum regions expressed both RAR and RXR. This result suggests that the formation of a RAR/RXR heterodimer in the presence of RA may play a physiological role in the growth and differentiation of gastric mucosa. Alteration in levels of RA or nuclear retinoid receptors may perturb cell growth or differentiation

© Cancer Research Campaign 1999

and may have an impact on gastric carcinogenesis. This is supported by the finding that patients with gastric cancer have lower levels of serum vitamin A than normal controls (Miasoedov et al, 1989), and that individuals with low serum β-carotene levels have an increased risk for the development of gastric cancer (Haenszel et al, 1985). Among the five receptor subtypes, RXRα mRNA appeared to express at the highest levels in various cell types of gastric mucosa. RXR can regulate gene expression through the formation of a RXR/RXR homodimer in response to 9-*cis* RA (Zhang et al, 1992). The receptor can also mediate signal transduction of retinoids, thyroid hormone and vitamin D through heterodimer formation between RXR and RAR, thyroid hormone receptor or vitamin D receptor (Lablanc and Stunnenbery, 1995). In addition, other receptors such as peroxisome proliferative activating receptor and orphan receptors are known to form heterodimers with RXR. Due to generally higher levels of RXRα expression than RAR in normal gastric mucosa, hormones such as thyroid hormone or vitamin D may, therefore, also play a physiological role in gastric mucosa.

The expression of RAR and RXR subtypes in normal and cancer tissues has been investigated in various tissues. Expression of RARα is decreased in tissues of squamous cell carcinoma (Issing and Wustrow, 1996) and mouse skin tumours (Darwiche et al, 1995, 1996), and the decrease is associated with activation of the protein kinase C or H-*ras* oncogene in mouse skin tumours (Darwiche et al, 1996). RARβ expression is decreased in tissues of the head and neck (Xu et al, 1994*a*), breast (Xu et al, 1997*b*; Widschwendter et al, 1997), larynx (Castillo et al, 1997) and non-small-cell lung cancers (Xu et al, 1997*a*). A decrease in RARγ expression is found in mouse skin tumours (Darwiche et al, 1995, 1996) and tissues of non-small-cell lung cancer (Xu et al, 1997*b*). An increase in RXRα and a decrease in RXRβ expression is found in tumours of skin (Darwiche et al, 1995) and non-small-cell lung cancer (Xu et al, 1997*a*) respectively. The difference in alteration of RAR and RXR subtypes among various tumours may be associated with tissue-specific functions of each retinoid receptor subtype as well as differences in mechanisms of carcinogenesis among tumours. In this study, we only observed a significant decrease in RARα expression in premalignant and malignant gastric tissues among the five investigated nuclear retinoid receptors. Mutation of K- and H-*ras* genes is detected, but uncommon, in some cancer tissues and is rarely detected in precancerous lesions (Soman et al, 1991; Tsuchiya et al, 1997). However, overexpression of K- and H-*ras* proteins are observed much more frequently in both premalignant and malignant gastric tissues (Czerniak et al, 1989). It is therefore possible that the decrease in RARα expression observed in gastric tissues may be related to the activation of the *ras* oncogene as observed in mouse skin tumours (Darwiche et al, 1996). However, *ras* mutation or overexpression is preferentially associated with the development of the well-differentiated type of gastric cancer (Tahara, 1993; Yoshida et al, 1988), which is inconsistent with the decrease in RARα expression in poorly differentiated gastric cancer with signet-ring cell type, as observed in this study. It is therefore likely that other genetic alterations, in addition to *ras* or PKC, may also contribute to the abnormal RARα expression in gastric tissues.

Our study observed a decrease in RARα expression in both premalignant and malignant gastric tissues. Similarly, a decrease in RARα, RARβ or RARγ expression is observed in premalignant tissues of the breast, oral, head and neck and skin (Xu et al, 1994*a*; Darwiche et al, 1995, 1996; Lotan et al, 1995; Widschewendter et al, 1997). Furthermore, McGregor et al (1997) have shown that the loss of RARβ expression occurs during the transition from senescent to immortal phenotypes. These data suggest that the decrease or loss of expression of nuclear retinoid receptors may occur before neoplastic transformation. The alteration may be reversible. Treatment with 13-*cis* retinoic acid can restore RARβ expression in premalignant oral tissues, and the restoration is correlated with a clinical response (Lotan et al, 1995). Therefore, some of the cancer-preventive activities of retinoids are mediated, first, through restoring the expression of nuclear retinoid receptors and, second, through bringing back the growth and differentiation control by retinoids in precancerous lesions. *N*-4-hydroxyphenyl retinamide has been found to prevent the progression of gastric dysplasia into cancer (Han, 1993). Whether the effect is associated with restoration of RARα expression requires further study.

During the process of gastric carcinogenesis, telomere reduction leading to genomic instability appears to occur at the earliest step (Tahara, 1993). Alterations in genes such as K- and H-*ras*, *p53*, c-*met* and tpr-*met* occur later in precancerous lesions. Activation of c-*erbB* and c-*met* oncogenes and loss of expression of genes like cadherin, DCC, transforming growth factor β and type I receptor of transforming growth factor β are involved in the later stages of gastric cancer. We observed a decrease in RARα expression in intestinal metaplasia, dysplasia and cancer tissues. Abnormality in expression of RARα appeared to occur at a similar stage to that seen in genes such as K- and H-*ras*, *p53*, c-*met* and trp-*met*. Currently, the possible link between these genes and RAR has not been investigated and requires further study.

In summary, this study observed the expression of RAR and RXR in normal gastric tissues, indicating the physiological function of RA on gastric mucosa. The decrease in expression of RARα in premalignant and malignant gastric tissues suggests that RARα has a significant role during processes of gastric carcinogenesis. Investigating the mechanism of the loss of RARα expression may provide further insight regarding the mechanism of growth control of gastric cancer cells.

### ACKNOWLEDGEMENTS

The authors would like to thank Mr K-J Huang and Dr W-H Lee for help in preparing slide sections, Dr W-H Chen for helpful discussions, and Drs P Chambon, M Phafl and RM Evans for providing cDNA plasmids. The study is supported from the National Science Council NSC86-2314-B016-054 and NSC87-2314-B016-068, Taipei, Taiwan, Republic of China.

### REFERENCES

Badley JE, Bishop GA, St John T and Frelinger JA (1988) A simple, rapid method for the purification of poly A$^+$ RNA. *Biotechniques* **6**: 114–116

Brand NJ, Petkovich M, Krust A, Chambon P, de The H, Marchio A, Tiollais P and Dejean A (1988) Identification of a second human retinoic acid receptor. *Nature (Lond)* **332**: 850–853

Castillo L, Milano G, Santini J, Demard F and Pierrefite V (1997) Analysis of retinoic acid receptor β expression in normal and malignant laryngeal mucosa by a sensitive and routine applicable reverse transcription-polymerase chain reaction enzyme-linked immunosorbent assay method. *Clin Cancer Res* **3**: 2137–2142

Czerniak B, Herz F, Gorczyca W and Koss LG (1989) Expression of ras oncogene p21 protein in early gastric carcinoma and adjacent gastric epithelia. *Cancer* **64**: 1467–1473

Darwiche N, Celli G, Tennenbaum T, Glick AB, Yuspa SH and De Luca LM (1995) Mouse skin tumor progression results in differential expression of retinoic acid and retinoid x receptors. *Cancer Res* **55**: 2774–2782

© Cancer Research Campaign 1999 

Darwiche N, Scita G, Jones C, Rutberg S, Greenwald E, Tennenbaum T, Collins SJ, De Luca LM and Yuspa SH (1996) Loss of retinoic acid receptors in mouse skin and skin tumors is associated with activation of the *ras*$^{Ha}$ oncogene and high risk for premalignant progression. *Cancer Res* **56**: 4942–4949

Fujii T and Yokomori T (1991) A clinical study of vitamin A concerning auxiliary chemotherapies after operation of gastric cancer. *Gan To Kagaku Ryoho* **18**: 265–269

Haenszel W, Correa P, Lopez A, Cuello C, Zarama G, Zavala D and Fontham E (1985) Serum micronutrient levels in relation to gastric pathology. *Int J Cancer* **36**: 43–48

Hamada K, Gleason SL, Levi BZ, Hirschfeld S, Appella E and Ozato K (1989) H-2RIIP, a member of the nuclear hormone receptor superfamily that binds to both the regulatory element of major histocompatibility class I genes and the estrogen response element. *Proc Natl Acad Sci USA* **86**: 8289–8293

Han J (1993) Highlights of cancer chemoprevention studies in China. *Preventive Med* **22**: 712–722

Heyman RA, Mangelsdorf DJ, Dyck JA, Stein RB, Eichele G, Evans RM and Thaller C (1992) 9-*cis*-retinoic acid is a high affinity ligand for the retinoid x receptor. *Cell* **68**: 397–406

Houle B, Rochette-Egly C and Bradley WE (1993) Tumor-suppressive effect of the retinoic acid receptor beta in human epidermoid lung cancer cells. *Proc Natl Acad Sci USA* **90**: 985–989

Hu L, Crowe DL, Rheinwald JG, Chambon P and Gudas LJ (1991) Abnormal expression of retinoic acid receptors and keratin 19 by human oral and epidermal squamous cell carcinoma cell lines. *Cancer Res* **51**: 3972–3981

Issing WJ and Wustrow TPU (1996) Expression of retinoic acid receptors in squamous cell carcinomas and their possible implication for chemoprevention. *Anticancer Res* **16**: 2373–2378

Jiang S-Y, Shyu R-Y, Chen H-Y, Lee M M-S, Wu K-L and Yeh M-Y (1996) In vitro and in vivo growth inhibition of SC-M1 gastric cancer cells by retinoic acid. *Oncology* **53**: 334–340

Kaiser A, Herbst H, Fisher G, Koenigsmann M, Berdel WE, Riecken E-O and Rosewicz S (1997) Retinoic acid receptor β regulates growth and differentiation in human pancreatic carcinoma cells. *Gastroenterology* **113**: 920–929

Leblanc BP and Stunnenberg HG (1995) 9-*cis* retinoic acid signaling: changing partners canses some excitement. *Genes Dev* **9**: 1811–1816

Liang K-Y and Zeger SL (1986) Longitudinal data analysis using generalized linear models. *Miometrika* **73**: 13–22

Lotan R, Xu XC, Lippman SM, Ro JY, Lee JS, Lee JJ and Hong WK (1995) Suppression of retinoic acid receptor-β in premalignant oral lesions and its up-regulation by isotretinoin. *N Engl J Med* **332**: 1405–1410

McGregor F, Wagner E, Felix D, Soutar D, Parkinson K and Harrison PR (1997) Inappropriate retinoic acid receptor-β expression in oral dysplasias: correlation with acquisition of the immortal phenotype. *Cancer Res* **57**: 3886–3889

Miasoedov DV, V'iunitskaia LV, Chernukhina LA and Donchenko GV (1989) Blood vitamin A contents in patients with stomach cancer. *Voprosy Onkologii* **35**: 945–958

Mangelsdorf DJ, Ong ES, Dyck JA and Evan RM (1990) Nuclear receptor that identifies a novel retinoic acid response pathway. *Nature (Lond)* **345**: 224–229

Mangelsdorf DJ, Umesono K and Evan RM (1994) The retinoid receptors. In *The Retinoids*, Sporn MB, Roberts AB and Goodman DS (eds), pp. 319–349. Raven Press: New York

Petkovich M, Brand NJ, Krust A and Chambon P (1987) A human retinoic acid receptor which belongs to the family of nuclear receptors. *Nature (Lond)* **330**: 444–450

Shyu R-Y, Jiang S-Y, Huang T-C, Wu K-L, Roffler SR and Yeh M-Y (1995) Growth regulation by all-trans-retinoic acid and retinoic acid receptor messenger ribonucleic acids expression in gastric cancer cells. *Eur J Cancer* **31A**: 237–243

Soman NR, Correa P, Ruiz BA and Wogan GN (1991) The TPR-MET oncogenic rearrangement is present and expressed in human gastric carcinoma and precursor lesions. *Proc Natl Acad Sci USA* **88**: 4892–4896

Tahara E (1993) Molecular mechanism of stomach carcinogenesis. *J Cancer Res Clin Oncol* **119**: 265–272

Tsuchiya C, Ohshima S and Takahama M (1997) Detection of c-Ki-ras oncogene mutation in gastric adenomas with formalin-fixed, paraffin-embedded biopsy materials. *J Gastroenterol* **32**: 28–33

Watanabe H, Jass JR and Sabin LH (1989) Histological typing of esophageal and gastric tumors. In *World Health Organization International Histological Classification of Tumors*, pp. 20–26. Springer-Verlag: Berlin

Widschwendter M, Berger J, Daxenbichler G, Muller-Holzner E, Widschwendter A, Mayr A, Marth C and Zeimet AG (1997) Loss of retinoic acid receptor β expression in breast cancer and morphologically normal adjacent tissue but not in the normal breast tissue distant from the cancer. *Cancer Res* **57**: 4158–4161

Xu XC, Ro JY, Lee JS, Shin DM, Hong WK and Lotan R (1994*a*) Differential expression of nuclear retinoid receptors in normal, premalignant and malignant head and neck tissues. *Cancer Res* **54**: 3580–3587

Xu X-C, Clifford JL, Hong WK and Lotan R (1994*b*) Detection of nuclear retinoic acid receptor mRNA in histological tissue sections using nonradioactive in situ hybridization histochemistry. *Diagnostic Mol Pathol* **3**: 122–131

Xu XC, Sozzi G, Lee JS, Pastorino U, Pilotti S, Jurie JM, Hong WK and Lotan R (1997*a*) Suppression of retinoic acid receptor beta in non-small-cell lung cancer in vivo: implication for lung cancer development. *J Natl Cancer Inst* **89**: 624–629

Xu XC, Sneige N, Liu X, Nandagiri R, Lee JJ, Lukmanji F, Hortobagyi G, Lippmann SM, Dhingra K and Lotan R (1997*b*) Progressive decrease in nuclear retinoic acid receptor β messenger RNA levels during breast carcinogenesis. *Cancer Res* **57**: 4992–4996

Yoshida K, Hamatani K, Koide H, Ikeda H, Nakamura N, Akiyama M, Tsuchiyama H, Nakayama E and Shiku H (1988) Preparation of anti-ras Mr 21 000 protein monoclonal antibodies and immunohistochemical analyses on expression of ras genes in human stomach and thyroid cancers. *Cancer Res* **48**: 5503–5509

Zelent A, Krust A, Petkovich M, Kastner P and Chambon P (1989) Cloning of murine α and β retinoic acid receptors and a novel receptor γ predominantly expressed in skin. *Nature (Lond)* **339**: 714–717

Zhang XK, Lehmann JM, Hoffmann B, Dawson MI, Cameron J, Graupner G, Tran P and Pfhal M (1992) Homodimer formation of retinoid X receptor induced by 9-*cis* retinoic acid. *Nature (Lond)* **358**: 587–591

*© Cancer Research Campaign 1999*

## References

[PDF_00586] Badley J. E., Bishop G. A., St John T., Frelinger J. A. (1988). A simple, rapid method for the purification of poly A+ RNA.. Biotechniques.

[PDF_00588] Brand N., Petkovich M., Krust A., Chambon P., de Thé H., Marchio A., Tiollais P., Dejean A. (1988). Identification of a second human retinoic acid receptor.. Nature.

[PDF_00591] Castillo L., Milano G., Santini J., Demard F., Pierrefite V. (1997). Analysis of retinoic acid receptor beta expression in normal and malignant laryngeal mucosa by a sensitive and routine applicable reverse transcription-polymerase chain reaction enzyme-linked immunosorbent assay method.. Clin Cancer Res.

[PDF_00595] Czerniak B., Herz F., Gorczyca W., Koss L. G. (1989). Expression of ras oncogene p21 protein in early gastric carcinoma and adjacent gastric epithelia.. Cancer.

[PDF_00598] Darwiche N., Celli G., Tennenbaum T., Glick A. B., Yuspa S. H., De Luca L. M. (1995). Mouse skin tumor progression results in differential expression of retinoic acid and retinoid X receptors.. Cancer Res.

[PDF_00603] Darwiche N., Scita G., Jones C., Rutberg S., Greenwald E., Tennenbaum T., Collins S. J., De Luca L. M., Yuspa S. H. (1996). Loss of retinoic acid receptors in mouse skin and skin tumors is associated with activation of the ras(Ha) oncogene and high risk for premalignant progression.. Cancer Res.

[PDF_00607] Fujii T., Yokomori T. (1991). [A clinical study of vitamin A concerning auxiliary chemotherapies after operation of gastric cancer].. Gan To Kagaku Ryoho.

[PDF_00610] Haenszel W., Correa P., López A., Cuello C., Zarama G., Zavala D., Fontham E. (1985). Serum micronutrient levels in relation to gastric pathology.. Int J Cancer.

[PDF_00613] Hamada K., Gleason S. L., Levi B. Z., Hirschfeld S., Appella E., Ozato K. (1989). H-2RIIBP, a member of the nuclear hormone receptor superfamily that binds to both the regulatory element of major histocompatibility class I genes and the estrogen response element.. Proc Natl Acad Sci U S A.

[PDF_00617] Han J. (1993). Highlights of the cancer chemoprevention studies in China.. Prev Med.

[PDF_00619] Heyman R. A., Mangelsdorf D. J., Dyck J. A., Stein R. B., Eichele G., Evans R. M., Thaller C. (1992). 9-cis retinoic acid is a high affinity ligand for the retinoid X receptor.. Cell.

[PDF_00622] Houle B., Rochette-Egly C., Bradley W. E. (1993). Tumor-suppressive effect of the retinoic acid receptor beta in human epidermoid lung cancer cells.. Proc Natl Acad Sci U S A.

[PDF_00625] Hu L., Crowe D. L., Rheinwald J. G., Chambon P., Gudas L. J. (1991). Abnormal expression of retinoic acid receptors and keratin 19 by human oral and epidermal squamous cell carcinoma cell lines.. Cancer Res.

[PDF_00628] Issing W. J., Wustrow T. P. (1996). Expression of retinoic acid receptors in squamous cell carcinomas and their possible implication for chemoprevention.. Anticancer Res.

[PDF_00631] Jiang S. Y., Shyu R. Y., Chen H. Y., Lee M. M., Wu K. L., Yeh M. Y. (1996). In vitro and in vivo growth inhibition of SC-M1 gastric cancer cells by retinoic acid.. Oncology.

[PDF_00634] Kaiser A., Herbst H., Fisher G., Koenigsmann M., Berdel W. E., Riecken E. O., Rosewicz S. (1997). Retinoic acid receptor beta regulates growth and differentiation in human pancreatic carcinoma cells.. Gastroenterology.

[PDF_00637] Leblanc B. P., Stunnenberg H. G. (1995). 9-cis retinoic acid signaling: changing partners causes some excitement.. Genes Dev.

[PDF_00641] Lotan R., Xu X. C., Lippman S. M., Ro J. Y., Lee J. S., Lee J. J., Hong W. K. (1995). Suppression of retinoic acid receptor-beta in premalignant oral lesions and its up-regulation by isotretinoin.. N Engl J Med.

[PDF_00650] Mangelsdorf D. J., Ong E. S., Dyck J. A., Evans R. M. (1990). Nuclear receptor that identifies a novel retinoic acid response pathway.. Nature.

[PDF_00644] McGregor F., Wagner E., Felix D., Soutar D., Parkinson K., Harrison P. R. (1997). Inappropriate retinoic acid receptor-beta expression in oral dysplasias: correlation with acquisition of the immortal phenotype.. Cancer Res.

[PDF_00647] Miasoedov D. V., V'iunitskaia L. V., Chernukhina L. A., Donchenko G. V. (1989). Soderzhanie vitamina A v plazme krovi bol'nykh rakom zheludka.. Vopr Onkol.

[PDF_00655] Petkovich M., Brand N. J., Krust A., Chambon P. (1987). A human retinoic acid receptor which belongs to the family of nuclear receptors.. Nature.

[PDF_00658] Shyu R. Y., Jiang S. Y., Huang S. L., Chang T. C., Wu K. L., Roffler S. R., Yeh M. Y. (1995). Growth regulation by all-trans-retinoic acid and retinoic acid receptor messenger ribonucleic acids expression in gastric cancer cells.. Eur J Cancer.

[PDF_00662] Soman N. R., Correa P., Ruiz B. A., Wogan G. N. (1991). The TPR-MET oncogenic rearrangement is present and expressed in human gastric carcinoma and precursor lesions.. Proc Natl Acad Sci U S A.

[PDF_00665] Tahara E. (1993). Molecular mechanism of stomach carcinogenesis.. J Cancer Res Clin Oncol.

[PDF_00667] Tsuchiya C., Ohshima S., Takahama M. (1997). Detection of c-Ki-ras oncogene mutation in gastric adenomas with formalin-fixed, paraffin-embedded biopsy materials.. J Gastroenterol.

[PDF_00673] Widschwendter M., Berger J., Daxenbichler G., Müller-Holzner E., Widschwendter A., Mayr A., Marth C., Zeimet A. G. (1997). Loss of retinoic acid receptor beta expression in breast cancer and morphologically normal adjacent tissue but not in the normal breast tissue distant from the cancer.. Cancer Res.

[PDF_00680] Xu X. C., Clifford J. L., Hong W. K., Lotan R. (1994). Detection of nuclear retinoic acid receptor mRNA in histological tissue sections using nonradioactive in situ hybridization histochemistry.. Diagn Mol Pathol.

[PDF_00677] Xu X. C., Ro J. Y., Lee J. S., Shin D. M., Hong W. K., Lotan R. (1994). Differential expression of nuclear retinoid receptors in normal, premalignant, and malignant head and neck tissues.. Cancer Res.

[PDF_00687] Xu X. C., Sneige N., Liu X., Nandagiri R., Lee J. J., Lukmanji F., Hortobagyi G., Lippman S. M., Dhingra K., Lotan R. (1997). Progressive decrease in nuclear retinoic acid receptor beta messenger RNA level during breast carcinogenesis.. Cancer Res.

[PDF_00683] Xu X. C., Sozzi G., Lee J. S., Lee J. J., Pastorino U., Pilotti S., Kurie J. M., Hong W. K., Lotan R. (1997). Suppression of retinoic acid receptor beta in non-small-cell lung cancer in vivo: implications for lung cancer development.. J Natl Cancer Inst.

[PDF_00691] Yoshida K., Hamatani K., Koide H., Ikeda H., Nakamura N., Akiyama M., Tsuchiyama H., Nakayama E., Shiku H. (1988). Preparation of anti-ras Mr 21,000 protein monoclonal antibodies and immunohistochemical analyses on expression of ras genes in human stomach and thyroid cancers.. Cancer Res.

[PDF_00695] Zelent A., Krust A., Petkovich M., Kastner P., Chambon P. (1989). Cloning of murine alpha and beta retinoic acid receptors and a novel receptor gamma predominantly expressed in skin.. Nature.

[PDF_00698] Zhang X. K., Lehmann J., Hoffmann B., Dawson M. I., Cameron J., Graupner G., Hermann T., Tran P., Pfahl M. (1992). Homodimer formation of retinoid X receptor induced by 9-cis retinoic acid.. Nature.

